# Epigenetic reprogramming by tumor-derived EZH2 gain-of-function mutations promotes aggressive 3D cell morphologies and enhances melanoma tumor growth

**DOI:** 10.18632/oncotarget.2758

**Published:** 2015-01-22

**Authors:** Anthony M. Barsotti, Michael Ryskin, Wenyan Zhong, Wei-Guo Zhang, Andreas Giannakou, Christine Loreth, Veronica Diesl, Maximillian Follettie, Jonathan Golas, Michelle Lee, Timothy Nichols, Conglin Fan, Gang Li, Stephen Dann, Valeria R. Fantin, Kim Arndt, Dominique Verhelle, Robert A. Rollins

**Affiliations:** ^1^ Oncology Research Unit, Pfizer Worldwide Research and Development, Pearl River, NY 10965, USA; ^2^ Oncology Research Unit, Pfizer Worldwide Research and Development, Cambridge, MA 02140, USA; ^3^ Oncology Research Unit, Pfizer Worldwide Research and Development, San Diego, CA 92121, USA; ^4^ Drug Safety Research and Development, Pfizer Worldwide Research and Development, San Diego, CA 92121, USA

**Keywords:** EZH2, EZH2 mutations, cancer epigenetics, melanoma, 3D culture, motility, Axonal guidance

## Abstract

In addition to genetic alterations, cancer cells are characterized by myriad epigenetic changes. EZH2 is a histone methyltransferase that is over-expressed and mutated in cancer. The EZH2 gain-of-function (GOF) mutations first identified in lymphomas have recently been reported in melanoma (~2%) but remain uncharacterized. We expressed multiple EZH2 GOF mutations in the A375 metastatic skin melanoma cell line and observed both increased H3K27me3 and dramatic changes in 3D culture morphology. In these cells, prominent morphological changes were accompanied by a decrease in cell contractility and an increase in collective cell migration. At the molecular level, we observed significant alteration of the axonal guidance pathway, a pathway intricately involved in the regulation of cell shape and motility. Furthermore, the aggressive 3D morphology of EZH2 GOF-expressing melanoma cells (both endogenous and ectopic) was attenuated by EZH2 catalytic inhibition. Finally, A375 cells expressing exogenous EZH2 GOF mutants formed larger tumors than control cells in mouse xenograft studies. This study not only demonstrates the first functional characterization of EZH2 GOF mutants in non-hematopoietic cells, but also provides a rationale for EZH2 catalytic inhibition in melanoma.

## INTRODUCTION

Cancer is a disease characterized by both genetic and epigenetic changes that disrupt tumor-suppressive pathways and activate tumor-promoting pathways. Perturbations of both DNA methylation and histone modification patterns resulting from mutations and altered expression of DNA and histone modifying enzymes have been observed in a wide variety of cancers [1, 2].

EZH2 is a histone methyltransferase that methylates (mono, di or tri) Lysine 27 of Histone H3, a mark that strongly correlates with gene repression [3]. During development, EZH2 acts within Polycomb Repressor Complex 2 (PRC2) to suppress developmental regulators (e.g. Hox proteins) and thus plays a role in establishing cellular identity [4]. In cancer, EZH2 is over-expressed in multiple tumor types including lung, prostate, breast, colon, bladder, and pancreatic cancer, as well as sarcoma, lymphoma, and melanoma. EZH2 expression often correlates with tumor aggressiveness and serves as an independent prognostic indicator of survival in lung, prostate, ovarian, breast and renal cell carcinomas [5–7]. In addition, hotspot mutations that activate EZH2 (e.g. Y641, A677, A687) have now been identified in hematological tumor types (including diffuse large B-cell lymphoma (DLBCL) and follicular lymphoma (up to 25%)) [8, 9].

The Y641 GOF mutations occur in a heterozygous fashion and alter the substrate specificity of EZH2 to dimethylated H3K27, thereby resulting in massive accumulation of H3K27me3 and depletion of H3K27me2 [10, 11]. Importantly, GOF mutations render DLBCL cell lines exquisitely sensitive to catalytic inhibition of EZH2 activity, suggesting that EZH2 activity is crucial for these tumor types and opening up the possibility of EZH2 inhibitor-based therapy for patients with these mutations [12–14]. Recently, a comprehensive analysis of driver mutations in melanoma has revealed the presence of EZH2 GOF mutations (2%) within this disease setting [15]. The role played by EZH2 GOF mutations in non-hematopoietic cells, and in particular melanoma cells, is completely uncharacterized. Studying the action of EZH2 GOF mutations in non-hematopoietic cells will allow both a greater understanding of EZH2 catalytic activity in solid tumors, as well as define and potentially expand the subset of patients who would benefit from EZH2-based therapies. In this study, we have utilized both ectopic expression of EZH2 GOF mutations and a small molecule inhibitor of EZH2 to gain insight into the function of EZH2 and EZH2 GOF mutants in solid tumor biology with a particular focus on melanoma.

## RESULTS

**Figure 1:**
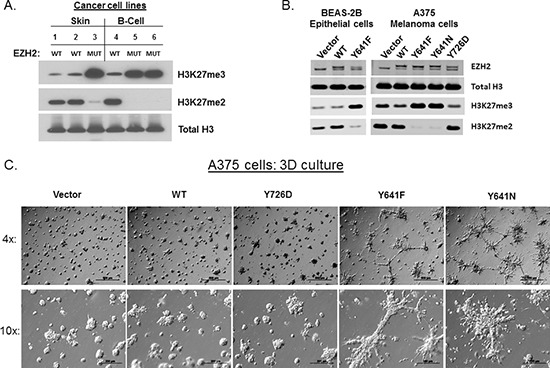
EZH2 GOF mutants greatly increase global H3K27me3 levels and cause 3D-culture phenotypes **(A)** H3K27me2/3 and Total H3 levels were probed by western blot from cell lines that contain either EZH2 WT or EZH2 GOF: 1.A431, 2.A375, 3.IGR1, 4.OCI-LY19, 5.KARPAS-422, 6.WSU-DL-CL2. **(B)** Western blot analysis following ectopic expression of EZH2 WT, GOF or LOF mutants in BEAS-2B epithelial cells or A375 melanoma cells. In lanes with ectopic EZH2, the upper band represents the ectopic EZH2 due to the presence of a V5 tag. **(C)** 4x and 10x images of A375 cells (expressing empty vector, EZH2 WT, EZH2 Y641F, EZH2 Y641N and EZH2 Y726D (LOF)) grown on top of a thick layer of ECM are shown.

To assess the impact of EZH2 GOF mutants on global levels of H3K27 modification, we performed western blotting from several cell lines that harbor endogenous EZH2 WT or EZH2 GOF mutants (Figure 1A). As reported, mutant-EZH2 DLBCL cells (Karpas-422 (lane 5), WSU-DL-CL2 (lane 6)) displayed increased H3K27me3 and depleted H3K27me2 compared to a WT-EZH2 DLBCL cell line (OCI-LY19 (lane 4)). Accordingly, the IGR1 melanoma cell line (lane 3) that contains an endogenous EZH2 Y641N mutation displayed a similar pattern of H3K27 modification compared to two WT-EZH2 skin cancer cell lines (A431 skin SCC (lane 1), A375 melanoma cells (lane 2)).

To evaluate the role of EZH2 GOF mutations, we introduced EZH2-expressing lentiviral constructs into two distinct cell models: A375 cells and BEAS-2B cells. A375 is a malignant skin melanoma cell line that harbors an activating BRAF mutation and was chosen based on the observation that EZH2 GOF mutations have been shown to occur concurrently with BRAF mutations in melanoma [16]. For comparison, a non-transformed epithelial cell line was utilized to assess the role of EZH2 GOF mutants in a normal genetic background. The western blot in Figure 1B shows that only EZH2 GOF mutants (Y641F, Y641N), but not EZH2 WT or a catalytically-inactive EZH2 point mutant (Y726D), caused increased H3K27me3 with subsequent decrease in H3K27me2 in both BEAS-2B and A375 cells. Importantly, this pattern of histone modification caused by ectopic EZH2 GOF mutants mirrors the pattern of cells with endogenous EZH2 mutations.

We next assessed the phenotypes that were associated with EZH2 GOF-dependent epigenetic reprogramming in the disease relevant A375 model. Despite the fact that GOF mutations are selected for within melanoma, the expression of EZH2 GOF mutants (Y641F, Y641N) gave no growth advantage to A375 cells grown under standard tissue culture conditions (Supplemental Figure S1A). Furthermore, there were no obvious changes to cell morphology in 2D culture (Supplemental Figure S1B). However, a clear phenotype became evident upon growing the cells in 3D culture. Strikingly, EZH2 GOF mutant-expressing cells seeded upon a thick layer of extracellular matrix (ECM) displayed prominent branching morphology compared to control cells (Vector), EZH2 WT and Y726D expressing cells (Figure 1C). EZH2 GOF-expressing cells embedded within ECM also showed a similar disruption of spheroid formation and aggressive growth morphology (Supplemental Figure S2A).

Upon observing these striking morphological changes, we asked whether these changes were specific to the expression of EZH2 Y641 mutants (the only GOF mutants observed thus far in melanoma), or whether enhancement of H3K27me3 levels in general could also give rise to these phenotypes. Therefore, we expressed two lymphoma-specific EZH2 GOF mutants, A677G and A687V, in A375 cells. Of note, these mutations have different substrate preferences compared to Y641 mutants (Supplemental Figure S2B, top panel). Nonetheless, the expression of both lymphoma-derived GOF mutants gave rise to increased global H3K27me3, but had distinct effects on H3K27me2 depletion. Despite its increased activity on H3K27me1 (with little gain in activity for H3K27me2), the observation that EZH2 A687V can drive conversion of H3K27me2 to H3K27me3 is in line with previous reports that have both predicted [17] and demonstrated this ability in cancer cell lines [18]. Despite the fundamental differences between these EZH2 GOF mutants, all GOF mutants gave rise to dramatic branching morphologies of A375 cells in 3D culture, thus strengthening the correlation between this phenotype and underlying epigenetic reprogramming (Supplemental Figure S2C).

While the images of cells grown in 3D-culture clearly demonstrate differences in cell morphology between control and EZH2 GOF-expressing cells, time-lapse imaging of these cells provides a more in-depth analysis of the behavior of such cells. A375 cells expressing an empty vector, EZH2 WT, EZH2 Y641F, EZH2 Y641N or EZH2 Y726D were cultured on top of ECM and images were recorded over a 96-hour period (Supplemental movie files 1–5). These data revealed that EZH2 GOF-expressing cells display enhanced motility compared to control cells, forming highly dynamic collective migrating chains instead of spheroid-like clusters that remained relatively stationary. Therefore, collective migration underlies the static branching morphology observed in EZH2 GOF-expressing cells shown in Figure 1.

To assess the generality of our findings, we expressed EZH2 WT or Y641F in non-transformed BEAS-2B cells and evaluated the resultant phenotypes. In this model, we observed that both EZH2 WT and Y641F disrupted the formation of spheroids typical of normal epithelium embedded within ECM (Supplemental Figure S3A). Notably, both EZH2 WT and Y641F also caused hallmarks of the epithelial to mesenchymal (EMT) transition. MosaiX image capture analysis (Supplemental Figure S3A, bottom panel, tiled images) revealed that control cells (Vector) formed E-cadherin positive spheroids that were disrupted by EZH2 WT and Y641F. Strikingly, EZH2 Y641F caused prominent branching growth of cells that stained positive for the mesenchymal marker Vimentin. Accordingly, both EZH2 WT and Y641F shifted the balance of the classic EMT markers E-Cadherin and Vimentin as well as other genes associated with this transition (Supplemental Figure S3B).

To identify the changes associated with the phenotypes described above, we performed microarray analysis on the BEAS-2B stable cell lines (Normalized expression values can be found in Supplemental Table S1). While EZH2 Y641F repressed a greater number of genes than EZH2 WT (Supplemental Figure S3C, note that numbers represent qualifiers), gene set enrichment analysis (GSEA) suggested that the both EZH2 WT and Y641F repressed genes from similar functional categories (Supplemental Figure S3D). In addition, the function of these gene sets corresponds well with the observed phenotypes. For example, EZH2 WT and Y641F both regulated gene sets associated with E-Cadherin (CDH1) and metastasis.

**Figure 2:**
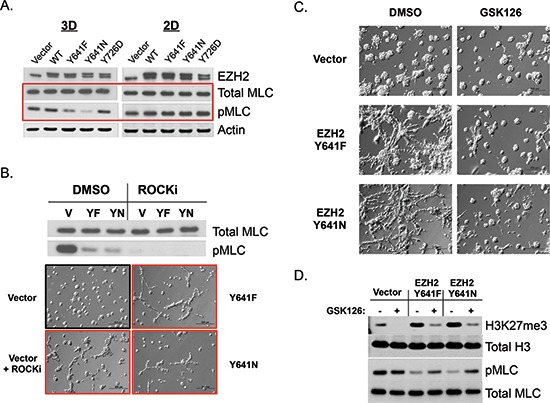
EZH2 GOF catalytic activity inhibits cell contractility and is necessary for the maintenance of 3D-branching morphology **(A)** Western blot analysis of A375 stable cells cultured on top of ECM (4 days) or tissue-culture treated plastic. pMLC is a marker of cell contractility and ROCK activity. **(B)** A375 Vector, EZH2 Y641F or EZH2 Y641N cells were seeded on top of ECM and simultaneously treated with 10 μM Y-27632 (ROCK inhibitor). Western blot analysis (top) was performed 4-days post-seeding/treatment. Images (10x, bottom) were captured 2 days post-seeding/treatment. **(C** and **D)** A375 stable cell lines were treated with DMSO or 1 μM GSK126 for 7 days in 2D culture and re-plated (and re-treated) on top of ECM and imaged (C, 10x) or lysed for western blot analysis (D) 4 days post-seeding.

While the 3D culture morphology changes caused by EZH2 in BEAS-2B epithelial cells can be attributed to the prominent EMT signature (including suppression of the master regulator E-Cadherin), A375 melanoma cells express very little to undetectable levels of E-Cadherin (not shown). While melanocytes are not of an epithelial cell lineage, they do express E-Cadherin in order to maintain contact with keratinocytes in the basal layer of the epidermis. In contrast, E-Cadherin is down-regulated in invasive or malignant melanoma cells (e.g. A375 cells) [19]. Therefore, we wished to understand how EZH2 GOF mutants caused branching morphogenesis and altered cell motility in the A375 cell model. We hypothesized that downstream regulation of the actin cytoskeleton had been altered in these cells. To understand these changes, we assessed the phosphorylation status of myosin light chain 2 (MLC2), a mark that correlates with myosin ATPase activity and contractility [20]. The western blot in Figure 2A demonstrates that phosphorylated myosin light chain 2 (pMLC) was greatly diminished in EZH2 GOF-expressing cells that had been cultured on a thick layer of ECM, but not under standard tissue culture conditions. Since pMLC is a marker of active Rho-associated protein kinase (ROCK), we treated A375 cells with Y-27632 (a well-characterized small molecule inhibitor of ROCK) to understand how ROCK activity may contribute to the observed phenotypes (Figure 2B). As in Figure 2A, pMLC levels were decreased by Y641F and Y641N GOF mutants. Expectedly, pMLC levels were efficiently depleted by treatment with the ROCK inhibitor. Two days after these cells were seeded onto ECM, ROCK-inhibited control cells displayed morphology and motility that was similar to EZH2 GOF-expressing cells (Figure 2B and Supplemental movie file 6). At later time-points, however, both ROCK inhibitor-treated Vector and EZH2 GOF cells formed extremely large cell clusters devoid of distinct branching structures (Supplemental Figure S4). Together these data suggest that while suppression of ROCK activity by EZH2 GOF mutants contributes to the observed 3D-morphology, this phenomenon is likely to require complex cellular organization that involves precise spatiotemporal regulation of signaling pathways.

We next wished to understand whether the changes in 3D morphology/motility caused by EZH2 GOF mutants were permanent or if they could be reversed by inhibition of EZH2. To address this question, we treated control and GOF-expressing cells with a previously published small molecule inhibitor of EZH2, GSK126 [12]. This treatment caused partial abrogation of the branching morphology of GOF expressing cells, restoring the spheroid-like morphology displayed by control cells (Figure 2C). Notably, at this time-point there were only minor effects of this inhibitor on control cells. (However, at later time-points control cells were also affected by EZH2 catalytic inhibition, Supplemental Figure S5A). Importantly, the EZH2 inhibitor caused robust down-regulation of H3K27me3, confirming the importance of EZH2 catalytic activity to 3D morphology changes. Furthermore, inhibition of the catalytic activity of EZH2 GOF mutants partially rescued pMLC levels, indicating that actin-myosin contractility was restored under these conditions (Figure 2D).

**Figure 3:**
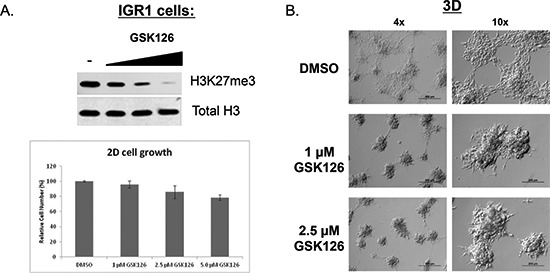
Catalytic inhibition of endogenous EZH2 Y641N (GOF) alters 3D-morphology of IGR1 melanoma cells **(A)** IGR1 cells were treated for 6 days with GSK126 at the indicated concentrations. Cells were counted or used to make lysates for western blot. Top panel: Western blot analysis of H3K27me3 levels following GSK126 treatment. Bottom panel: quantification of cell number (mean −/+ STDEV). **(B)** IGR1 cells were treated with DMSO, 1 μM or 2.5 μM GSK126 for 7 days in 2D culture and then re-plated (and re-treated) on top of ECM. Images (4x, 10x) were captured 6 days post-seeding.

To assess the therapeutic implications of our findings in the A375 model, we next extended our studies to the IGR1 melanoma cell line that contains an endogenous EZH2 GOF mutation (Y641N, see Figure 1A). Treatment of these cells with the EZH2 inhibitor decreased global H3K27me3 levels, but did not affect 2D cell growth up to a concentration of 2.5 μM, and had only minor effects on proliferation at 5 μM (Figure 3A). Remarkably, at concentrations that do not affect 2D cell growth, EZH2 catalytic inhibition caused significant changes in the 3D-morpholgy of IGR1 cells seeded upon a thick layer of ECM. Control cells (DMSO-treated) distributed evenly on the ECM, while EZH2 inhibitor-treated cells formed dense structures with little connectivity to neighboring cell clusters. Intriguingly, we also observed up-regulation of E-Cadherin in EZH2 inhibitor-treated IGR1 cells (Supplemental Figure S5B), suggesting that EZH2 may in regulate EMT in a subset of melanomas.

**Figure 4:**
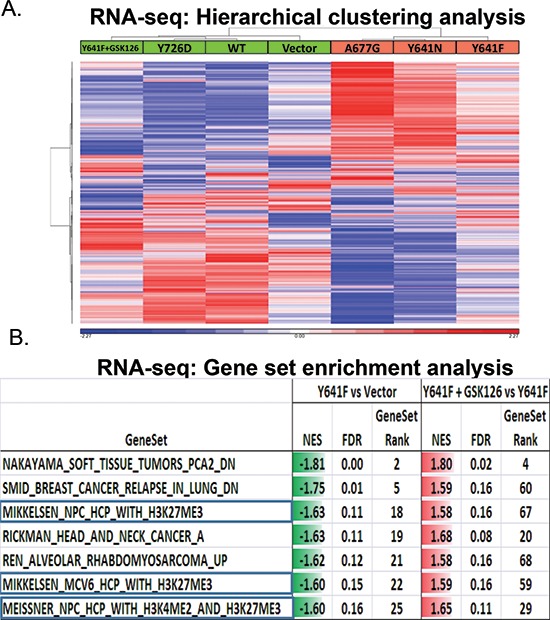
RNA-seq analysis of A375 stable cells reveals a correlation between EZH2 catalytic activity and global gene expression patterns **(A)** Hierarchical clustering analysis of mRNA expression levels of A375 stable cell lines, including A375 Y641F + GSK126 (left column), is displayed. Red color indicates high gene expression values and blue color indicates low gene expression values (standardized values). **(B)** A table containing the results from Gene Set Enrichment Analysis is displayed. Left column: A375 Y641F versus vector control cells. Right column: GSK126-treated A375 Y641F versus A375 Y641F.

To further understand the molecular underpinnings of the phenotypes described above, we assessed global levels of mRNA expression by performing RNA-sequencing (RNA-seq) from the following cell lines cultured on ECM: A375 Vector (control), A375 EZH2 WT, A375 Y726D (LOF), A375 Y641F (GOF), A375 Y641N (GOF), A375 A677G (GOF). To determine the impact of EZH2 catalytic inhibition on target gene expression, we also performed RNA-seq from A375 Y641F −/+ EZH2 inhibitor and IGR1 cells −/+ EZH2 inhibitor (FPKM values for the A375 and IGR1 RNA-seq can be found in Supplemental Table S2). Concordant with observed phenotypes in A375 cells, hierarchical clustering analysis showed that EZH2 GOF mutants had similar mRNA expression profiles that were distinct from control cells, EZH2 WT-expressing cells and EZH2 Y726D (LOF)-expressing cells (Figure 4A). Importantly, the gene expression pattern of inhibitor-treated EZH2 Y641F cells clustered together with control cells, suggesting that genes initially repressed by EZH2 Y641F were re-activated upon EZH2 catalytic inhibition. We looked at this phenomenon in greater detail by performing GSEA. This analysis revealed that compared to control cells, EZH2 Y641F down-regulated gene sets that have been previously found associated with H3K27me3 promoter modification. These gene sets were also de-repressed by catalytic inhibition of EZH2 (Figure 4B).

**Figure 5:**
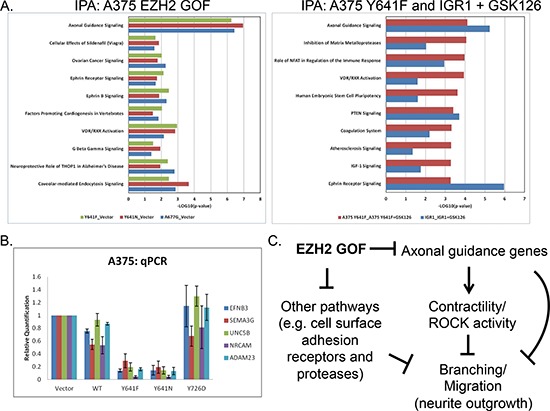
EZH2 GOF mutants regulate axonal-guidance genes **(A)** Ingenuity Pathway Analysis was performed from RNA-seq data of A375 stable cells (left panel) as well as GSK126-treated A375 Y641F and IGR1 cells (right panel). Pathways that were commonly regulated with high statistical significance (*p*-value < = 0.05) by all EZH2 GOF mutants or GSK126-treatment are shown. Greater –LOG(*p*-value) values indicates greater statistical significance of pathway enrichment. **(B)** qPCR was used to validate gene expression changes observed by RNA-seq (mean −/+ STDEV). **(C)** A model of EZH2 GOF-dependent pathway regulation leading to observed 3D-phenotypes.

Next, we utilized Ingenuity Pathway Analysis (IPA) to understand the functional cellular networks impacted by EZH2 GOF expression (Figure 5A). The axonal guidance pathway represents one such pathway that was commonly regulated by all three GOF mutants with high statistical significance. This functional category contains well-characterized gene families (e.g. Ephrins, Semaphorins, etc.) that direct neurite outgrowth or retraction (Supplemental Figure S6A). IPA analysis of both EZH2 inhibitor-treated IGR1 cells and A375 EZH2 Y641F cells also revealed EZH2-dependent regulation of genes involved in axonal guidance (as well as Ephrin receptor signaling), suggesting that the A375 GOF-expressing cells recapitulate endogenous functions of EZH2 GOF mutations in melanoma. Intriguingly, many of the suppressed genes are thought to provide repulsive signals to migrating neurons. In addition, the expression of cell surface adhesion receptors and proteases involved in axonal guidance were also regulated by EZH2 GOF mutants. We selected several such genes for confirmation by qRT-PCR analysis (Figure 5B, Supplemental Figure S6B).

A comprehensive view of the *in vitro* data presented in this manuscript point toward a model in which EZH2 GOF mutants alter both ECM adhesion and actin dynamics to promote branching morphology and collective migration in 3D culture (Figure 5C). Specifically, EZH2 GOF mutants repress axonal guidance genes and other gene sets (e.g. cell surface adhesion receptors and proteases) and alter the way in which these cells interact with the ECM and with themselves. These changes likely lead to differential intracellular signaling such as the inactivation of ROCK, thereby curbing its ability to cause contractility and neurite retraction.

**Figure 6:**
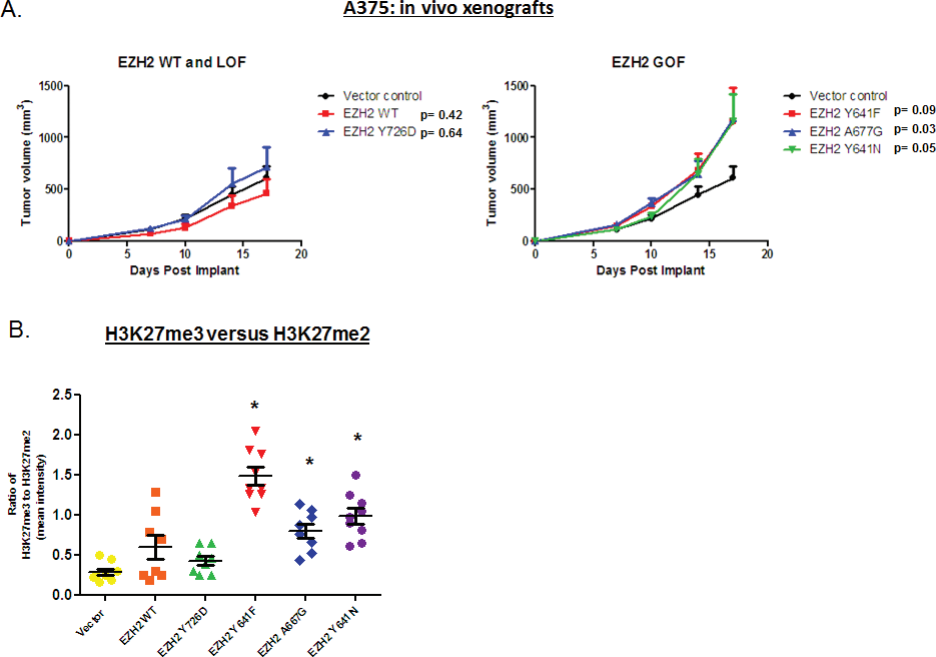
A375 cells expressing EZH2 GOF mutants (but not WT or LOF mutant) display increased tumor volume in mouse xenograft models A375 cells were subcutaneously implanted into nude mice and tumor volume was recorded over a period of 17 days. Data points represent mean tumor volume at each time-point. A two-tailed t-test performed for mean volumes at day 17 was used to calculate significance values. **(A)** Xenograft growth curves for Vector (control, black), EZH2 WT (red) and EZH2 Y726D (blue) are displayed (left panel). Xenograft growth curves for Vector (control, black), EZH2 Y641F (red), EZH2 Y641N (green), EZH2 A677G (blue) are displayed (right panel). **(B)** A graph depicting the ratio of mean intensity of H3K27me3 to H3K27me2 for each group is shown.

Finally, we wished to understand the contribution of these changes to *in vivo* tumor growth. We subcutaneously implanted 1 × 10^6^ A375 Vector (control), A375 EZH2 WT, A375 Y726D (LOF), A375 Y641F (GOF), A375 Y641N (GOF), or A375 A677G (GOF) cells into nude mice. Tumor volume was recorded over a period of 17 days. As shown in Figure 6A, A375 control xenografts grew rapidly, achieving a mean tumor volume of 608.46 mm^3^ by day 17. The expression of EZH2 WT or EZH2 Y726D did not impart any significant growth advantage or disadvantage to A375 xenografts (left panel). However, all three EZH2 GOF mutants provided a significant growth advantage to A375 xenografts, as EZH2 GOF-expressing cells formed tumors with a mean volume of ~1150 mm^3^ by day 17 (right panel). Using immunohistochemistry (IHC), we assessed whether the epigenetic pattern of H3K27 modification was maintained in xenografts. Figure 6B demonstrates that EZH2 GOF-expressing cells displayed significantly increased mean ratios of H3K27me3 to H3K27me2 intensity compared to control cells. From these studies we conclude that EZH2 GOF mutants maintain enhanced histone methyltranferase activity *in vivo* and that this activity correlates with increased tumor growth.

## DISCUSSION

In this study we have examined the putative functions of EZH2 GOF mutants in both melanoma cells and non-tumorigenic cells. We have learned that in both systems EZH2 GOF mutations greatly increased H3K27me3 levels, down-regulated a multitude of genes and caused striking 3D morphology changes. In epithelial cells, we observed an EZH2-induced EMT, a process associated with increased metastatic potential that has been previously been linked to EZH2 activity [21–23]. In this non-tumorigenic model, both EZH2 WT and Y641F caused morphological changes to cells in 3D culture. In contrast, we have found that only exogenously expressed EZH2 GOF mutants, but not EZH2 WT (or LOF), caused changes to 3D morphology in A375 melanoma cells. Most importantly, it was this activity (and not proliferation rate) that correlated with enhanced tumor growth as only EZH2 GOF expressing cells displayed increased A375 tumor size in xenograft studies. This finding underscores the need for a deeper understanding of EZH2 function within this disease setting.

The observation that four distinct EZH2 GOF mutants, that each lead to increased global H3K27me3, caused similar 3D morphologies provides compelling evidence for the specificity and relevance of this phenotype. The abrogation of branching morphology by a small-molecule inhibitor of EZH2 reaffirms the importance of EZH2 catalytic activity in this model. The observation that EZH2 inhibitor-treated IGR1 cells displayed a similar morphological reversion in 3D culture points toward conserved functions of EZH2 GOF mutants across melanoma cell lines. Unlike DLBCL cell lines that harbor endogenous EZH2 GOF mutants, IGR1 cell lines were insensitive to EZH2 catalytic inhibition in 2D culture. Interestingly, a recent study showed that ovarian cancer cells were insensitive to EZH2 inhibition in 2D culture, but sensitive to such treatment in 3D culture [24]. Taken together, these data suggest that the EZH2 GOF mutations may not drive intrinsic melanoma cell proliferation (as do mutations in driver genes like BRAF), but rather function by shaping the interactions between melanoma cells and their microenvironment. In line with this idea, previous clinical data have demonstrated an association between EZH2 levels and aggressive features of cutaneous melanoma [25]. Furthermore, as EZH2 mutations in melanoma are coincident with BRAF mutations they may serve to complement the function of BRAF (i.e. BRAF signaling drives cell division, whereas EZH2 signaling drives motility/migration). Pointing toward collaboration of these two oncogenes is the observation that knockdown of BRAFV600E (in A375 cells and other cell models) leads to down-regulation of EZH2 levels [26]. As such, the simultaneous inhibition of both BRAF and EZH2 should be considered in treatment of melanoma patients that harbor these mutations.

RNA-seq analysis from cells cultured in 3D allowed a thorough understanding of the changes imparted by EZH2 GOF mutations. The conserved pattern of gene expression between EZH2 GOF mutants strongly argues for a common mechanism underlying the observed phenotypes. Axonal guidance genes represent factors that control neuronal migration and positioning by providing short-range (cell surface localization) or long-range (secreted/diffusible) attractive or repulsive cues [27]. Factors that regulate axonal-guidance include, but are not limited to, gene families like Ephrins, Semaphorins, Netrins, SLITs, and ROBOs as well as ECM components, cell adhesion molecules and cell-surface proteases. These factors are also thought to be crucial players in the migration of neural crest-derived melanocytes [28–30]. It is also well-established that signaling through this pathway converges on the Rho-GTPase family that acts as a master regulator of neurite outgrowth [31, 32] and cell motility [33]. For example, Eph-receptor-ephrin bi-directional signaling greatly contributes to regulation of cell morphology and motility through functional interactions with Rho GTPases [34, 35].

Therefore, we hypothesize that the coordinate regulation of this signaling pathway by EZH2 GOF mutants contributes to the observed collective migration and branching morphology in A375 melanoma cells. In support of this hypothesis, many of the suppressed genes are thought to be negative regulators of axon-growth. For example, Ephrin-B3 has been shown to both inhibit neurite outgrowth and prevent regeneration after injury [36, 37]. Semaphorins can also provide repulsive forces to growing axons through their interaction with Neuropilin and Plexin receptors [38]. Similarly, the netrin receptor Unc5B inhibits both axonal migration as well as sprouting angiogenesis [39, 40]. Accordingly, axonal guidance genes have also been linked to the process of tumor angiogenesis [41].

Further implicating the axonal guidance genes in cancer biology are studies that have shown genetic alterations and aberrant regulation of these genes in several tumor types. For example, a recent genomic analysis of pancreatic ductal adenocarcinoma (PDAC) identified recurrent mutations and altered copy number of axonal guidance genes, implying a functional role in PDAC [42]. In addition, altered expression levels of axonal guidance genes have been observed in lung, breast and colorectal cancers, among others [43, 44]. Most relevant to this study, oncogenic BRAF mutants have been shown to down-regulate the expression of axonal guidance genes in melanoma [45]. Finally, many of the genes down-regulated by EZH2 GOF in this study that modulate ECM-adhesion/signaling display altered expression or have been ascribed roles in tumor biology (e.g. NRCAM, CEACAM1, SORCS1, ADAM23, MME).

Overall this study suggests that the gene expression changes resulting from EZH2 GOF expression have altered the interaction between cells and the ECM in two distinct cell models representing both non-tumorigenic and tumorigenic settings. Specifically in melanoma, we have delineated a novel pathway regulated by both ectopically and endogenously expressed EZH2 GOF mutants: the axonal guidance pathway. The 3D-culutre phenotypes that we have observed are likely caused by the cumulative effect of EZH2 GOF-dependent epigenetic reprogramming that results in the altered expression of multiple genes in this pathway (axonal guidance) as well as other pathways (adhesion, protease activity, etc.). Consequently, the alterations to these pathways have impacted intracellular signaling pathways, exemplified here by ROCK activity, that control cell motility and morphology. Though future studies will be needed to assess the metastatic capabilities of EZH2 GOF expressing cells, these cells indeed formed larger tumors than control cells in xenograft models. As these mutations do not enhance 2D-growth rate and the A375 melanoma cell line is a model that already grows rapidly *in vivo*, these surprising findings suggest that EZH2 GOF mutants likely impart properties to these cells that go beyond driving proliferation. Taken together, the data presented in this study strongly suggest that EZH2 GOF mutants contribute to melanoma progression by altering the way in which melanoma cells interface with their surrounding microenvironment and in this manner may confer a growth advantage to tumors bearing these mutations. This study supports an important role for EZH2 catalytic activity in melanoma and warrants further study of the utility of EZH2 inhibitors within this disease setting.

## METHODS

### Cell culture

Two suppliers of ECM were used: BD biosciences (RGF Matrigel^TM^, 356231) or Life Technologies (Geltrex^TM^, A1413302). To perform 3D culture experiments, 300 μl of ECM was used to coat one well of a 24-well plate and cells were seeded on top. For embedded 3D-culture experiments, cells were suspended in 100% ECM and plated into a 24-well plate. To obtain cell lysates from 3D culture experiments, the Cultrex® 3D-cell harvesting kit from Trevigen was utilized (3448–020-K). The GSK126 EZH2 inhibitor was obtained from Xcessbio (M60071) and used at a concentration of 1 μM unless otherwise noted. For 3D culture experiments involving the EZH2 inhibitor, cells were pre-treated in 2D for seven days prior to plating in 3D culture (and re-treated at the time of plating). The ROCK inhibitor Y-27632 was obtained from Sigma (Y0503) and was used at a concentration of 10 μM. For 3D culture experiments, this compound was added as cells were seeded onto the ECM.

### Microscopy

For imaging, an Olympus IX51 inverted microscope and DP71 camera was utilized.

### Western blotting

Antibodies: EZH2 (Cell Signaling Technology 3147), Actin (MAB1501), Total Histone H3 (Cell Signaling Technology 3638), H3K27me3 (Cell Signaling Technology 9733), H3K27me2 (Cell Signaling Technology 9728), MLC2 (Cell Signaling Technology 3672), pMLC2 (ser 19) (Cell Signaling Technology 3671), E-Cadherin (BD Biosciences 610182), and Vimentin (Epitomics 4211–1).

### qRT-PCR

The Qiagen RNeasy® kit (74106) and Qiagen Quantitect® Reverse Transcription kits (205311) were used to generate cDNA used in qPCR reactions (Applied Biosystems® ViiA 7^TM^ real-time cycler) in conjunction with Taqman gene expression master mix (4369016) and TaqMan gene expression assays.

### BEAS-2B gene expression profiling

BEAS-2B stable cells were seeded into 6-well plates in triplicate and 72 post-plating RNA was isolated using the Qiagen RNeasy® kit (74106). RNA was labeled and hybridized onto Affymetrix Human Genome U133 Plus 2.0 oligonucleotide microarrays arrays according to the manufacturer's instructions (Affymetrix). The gene expression data was processed by Micro Array Suite 5.0 (MAS5) algorithm followed by quantile normalization. Gene Set Enrichment analysis was conducted using the Gene Set Enrichment Analysis (GSEA) software (Broad Institute) [46].

### A375 and IGR1 RNA-Seq analysis

50bp paired-end reads were aligned to human genome (hg19) using RSEM software package [47]. Differential analysis was conducted using DESeq R package [48] with the expected counts produced by RSEM. Hierarchical clustering was carried out with Genomic Suite (Partek) using genes with max RPKM > = 5 in at least one sample. Differentially expressed genes were filtered by fold change > = 1.5 or fold change < = −1.5 and with *p*-value < = 0.05. Gene Set Enrichment analysis was conducted using the Gene Set Enrichment Analysis (GSEA) software (Broad Institute) with GseaPreranked option. Pathway analysis was conducted on differentially expressed genes using Ingenuity Pathway Analysis (IPA) software. Enriched gene sets were determined using Fisher Exact Test, and those that are common to all three GOF mutations with *p*-value < = 0.05 were compared and ranked by the %CV of the enrichment *p*-value.

### *In vivo* mouse xenografts

Female Nu/Nu (NU-Foxn-1, 6–8-week, Charles River) mice were used for all *in vivo* studies. All animal experiments were conducted in compliance with federal and institutional animal care and use committee requirements. Tumor cells were supplemented with 50% Matrigel^TM^ (BD Biosciences) to facilitate tumor take and growth as xenografts. 1 × 10^6^ (200 μl) of A375 Vector, A375 EZH2 WT, A375 EZH2 Y726D, A375 EZH2 Y641F, A375 EZH2 A677G, and A375 EZH2 Y641N were implanted s.c. into the hind flank region of the mouse. Mice were weighed and tumors measured with calipers twice weekly until the end of the study.

For further details, please see the Supplemental Experimental Procedures section.

## SUPPLEMENTAL MATERIALS AND METHODS FIGURES AND TABLES






