# Supplementary material for: Epigenetic reprogramming by tumor-derived EZH2 gain-of-function mutations promotes aggressive 3D cell morphologies and enhances melanoma tumor growth
Source: Oncotarget. 2015 Jan 22;6(5):2928–38. doi: 10.18632/oncotarget.2758 (PMC4413628; doi:10.18632/oncotarget.2758)
Supplement: Supplementary file 1 [file oncotarget-06-2928-s001.pdf]

SUPPLEMENTAL MATERIALS AND METHODS

Cell culture

BEAS-2B cells (immortalized lung epithelial cells [1], A375 cells (melanoma) and A431 (skin SCC) were obtained from the American Type Tissue Culture Collection (ATCC). IGR1 cells (melanoma), Karpas-422 (DLBCL), WSU-DL-CL2 (DLBCL) and OCI-LY19, (DLBCL) cells were obtained from the Leibniz Institute DSMZ. BEAS-2B cells were maintained in BEGM media (BEGM Bulletkit™ from Lonza (CC-3170). A375 cells were maintained in DME supplemented with 10% FBS, Sodium Pyruvate, Non-essential amino acids and L-Gutamine. IGR1 cells were maintained in RPMI supplemented with 10% FBS.

Lentiviral constructs expressing EZH2 WT or GOF mutants were used to derive stable cell lines. Wild-type EZH2 was cloned into the pLenti 6.3/TO/V5 (Life Technologies) vector backbone to generate lentivirus and Quickchange site-directed mutagenesis (Strategene) was used to derive point mutations in EZH2. These lentiviruses were used to infect either BEAS-2B or A375 cells. Cells were selected with Blasticidin HCL (Life Technologies, A11139); BEAS-2B: 0.5 mg/ml, A375: 5 mg/ml. For quantification of 2D proliferation, cell number was quantified directly using the ViCell-XR (Beckman Coulter) or indirectly using the Cell Titer Glo® reagent (Promega G7573).

Microscopy

For 4x and 10x images of cells, an Olympus IX51 inverted microscope (relief contrast) and DP71 camera/ DP Controller software was utilized. For time-lapse cell imaging (96 hours) and MosaiX image capture, Axiovision 4.8 software was used in conjunction with a Zeiss Axio Observer Z1 inverted microscope. Standard immunofluorescence protocols were used for 2D-culture, and for 3D-cultures the protocol was adapted from Lee et al [2]. The following antibodies were used for IF: E-Cadherin (BD Biosciences 610182), Vimentin (Epitomics 4211–1).

Western blotting

Cells from 2D or 3D culture were lysed in NuPAGE® LDS buffer (NP0007) and sonicated. Lysates were run using the Novex® NuPAGE® SDS-Page Gel System and blotted using standard procedures. Detection was done using either the ODYSSEY® system (LICOR) or using ECL (GE Healthcare). The following antibodies were used: EZH2 (Cell Signaling Technology 3147), Actin (MAB1501), Total Histone H3 (Cell Signaling Technology 3638), H3K27me3 (Cell Signaling Technology 9733), H3K27me2 (Cell Signaling Technology 9728), MLC2 (Cell Signaling Technology 3672), pMLC2 (ser 19) (Cell Signaling Technology 3671), E-Cadherin (BD Biosciences 610182), and Vimentin (Epitomics 4211–1).

qRT-PCR

RNA was isolated using the Qiagen RNeasy® kit (74106) and reverse transcription was performed using the Qiagen Quantitect® Reverse Transcription kit (205311). cDNA was used in qPCR reactions run on the Applied Biosystems® ViiA 7™ real-time cycler using Taqman gene expression master mix (4369016) in conjunction TaqMan gene expression assays: CDH1 (Hs01023894_m1), DSC3 (Hs00170032_m1), DSG3 (Hs00951897_m1), ESRP1 (Hs00214472_m1), EFNB3 (Hs00154861_m1), SEMA3G (Hs00220101_m1), UNC5B (Hs00900710_m1), NRCAM (Hs01031598_m1), ADAM23 (Hs00187022_m1), EPHB3 (Hs00177903_m1), KALRN (Hs00610200_m1), SEMA6B (Hs00220339_m1), CEACAM1 (Hs00989786_m1), SORCS1 (Hs00364666_m1). RPN1 (Hs00161446_m1) was used for normalization.

A375 and IGR1 RNA-Seq

RNA-seq was performed in conjunction with Expression Analysis, Inc.:

Libraries are prepared for RNA-Seq using the TruSeq RNA Sample Prep Kit (Illumina), including the use of Illumina in-line control spike-in transcripts. Prior to library preparation, RNA samples are quantitated by spectrophotometry using a Nanodrop ND-8000 spectrophotometer, and assessed for RNA integrity using an Agilent 2100 BioAnalyzer or Caliper LabChip GX. RNA samples with A260/A280 ratios ranging from 1.6 – 2.2, with RIN values ≥ 7.0, and for which at least 500 ng of total RNA is available will proceed to library preparation.

Library preparation begins with 500 ng of RNA in 50 µl of nuclease-free water, which is subjected to poly(A)+ purification using oligo-dT magnetic beads. After washing and elution, the polyadenylated RNA is fragmented to a median size of ~ 150 bp and then used as a template for reverse transcription. The resulting single-stranded cDNA is converted to double-stranded cDNA, ends are repaired to create blunt ends, then a single A residue is added to the 3' ends to create A-tailed molecules. Illumina indexed sequencing adapters are then ligated to the A-tailed double-stranded cDNA. A single index is used for each sample. The adapter-ligated cDNA is then subjected to PCR amplification for 15 cycles. This final library product

is purified using AMPure beads (Beckman Coulter), quantified by qPCR (Kapa Biosystems), and its size distribution assessed using an Agilent 2100 BioAnalyzer or Caliper LabChip GX. Following quantitation, an aliquot of the library is normalized to 2 nM concentration and equal volumes of specific libraries are mixed to create multiplexed pools in preparation for sequencing.

Immunohistochemistry (IHC)

Xenograft tumor tissue samples were received fixed in 10% Neutral Buffered Formalin, processed, and embedded. FFPE sections were taken at 4 micron thickness.

Immunofluoresence (IF) simultaneous multiplex staining technique was performed on the same FFPE sections using a manual platform. After dewax, antigen retrieval in Diva Decloaker (Biocare, #DV2004MX) and blocking, the following antibodies; H3me2k27 (1:400, Cell Signaling Tech, #9728) and H3me3k27 (1:100, Abcam, #ab6147) were cocktailed and incubated overnight in 4C. The sections were then incubated with Alexa Fluor Secondaries and coverslipped with fluorescent mounting media containing DAPI.

For mean H3K27me3/2 intensity quantification, up to 40–20x representative images per slide were multispectrally captured on the CRi Vectra™ system. Using Nuance™, appropriate spectral libraries were created and background was thresholded from each image. Samples were evaluated using Inform™ software and an algorithm classified by functional regions (Viable tumor, non-tumor, and background). Graphpad and Excel were used to further evaluate the numerical data generated by Inform. All statistical analyses were completed using a 2-tailed *T* test within GraphPad Prism.

REFERENCES

1. Reddel RR, Ke Y, Gerwin BI, McMenamin MG, Lechner JF, Su RT, Brash DE, Park JB, Rhim JS, Harris CC. Transformation of human bronchial epithelial cells by infection with SV40 or adenovirus-12 SV40 hybrid virus, or transfection via strontium phosphate coprecipitation with a plasmid containing SV40 early region genes. Cancer research. 1988; 48:1904–1909
2. Lee GY, Kenny PA, Lee EH, Bissell MJ. Three-dimensional culture models of normal and malignant breast epithelial cells. Nature methods. 2007; 4:359–365

SUPPLEMENTARY FIGURES AND TABLES

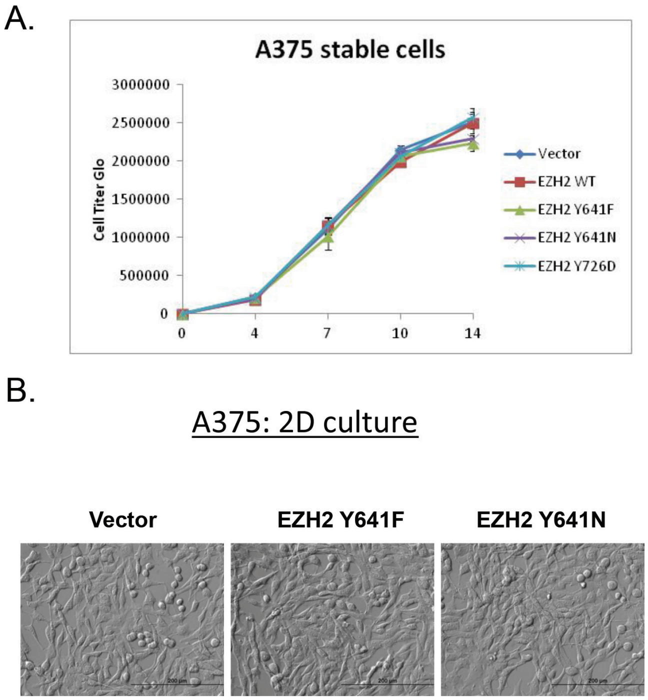

Supplementary Figure S1: EZH2 GOF mutants do not alter 2D growth properties of A375 melanoma cells. (A) The growth of A375 stable cells lines was assessed on indicated days by the use of Cell Titer Glo. **(B)** 10x images of A375 Vector, EZH2 Y641F and Y641N (GOFs) grown in standard 2D-culture are displayed.

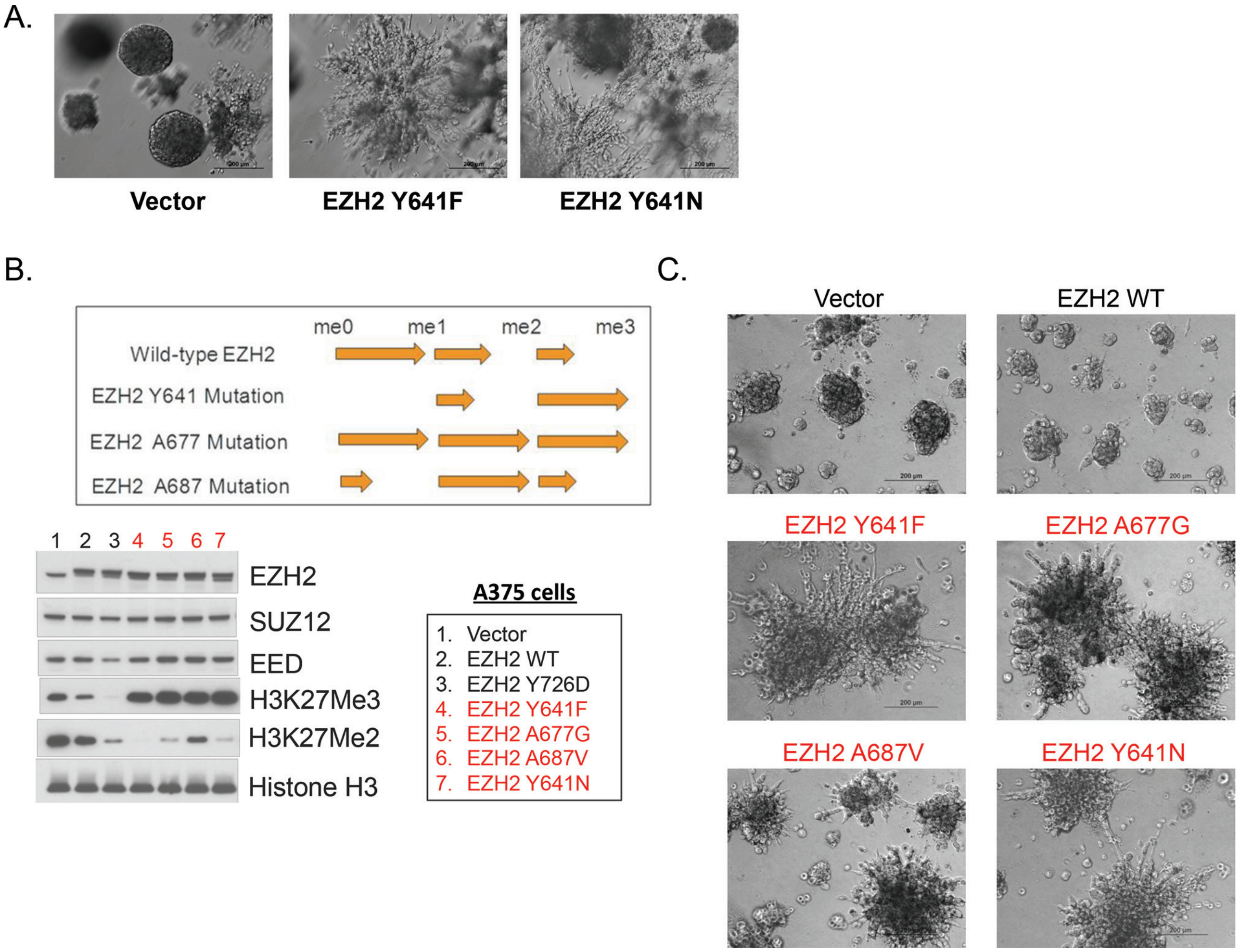

Supplementary Figure S2: Additional lymphoma-derived EZH2 GOF mutants cause increased global H3K27me3 and alter 3D-morphology in A375 cells. **(A)** A375 Vector (control), EZH2 Y641F and Y641N expressing cells were embedded within ECM. Images (10x) were captured 10 days post-seeding. **(B)** The chart shows the biochemical activity of each version of EZH2 toward unmodified or modified H3K27 peptides. The western blot below shows the global changes in H3K27me2/3 levels in A375 cells following the ectopic expression of these various EZH2 constructs (EZH2 GOF mutants are highlighted in red). **(C)** The 3D morphology of these A375 stable cell lines is displayed (EZH2 GOF mutants are again highlighted in red).

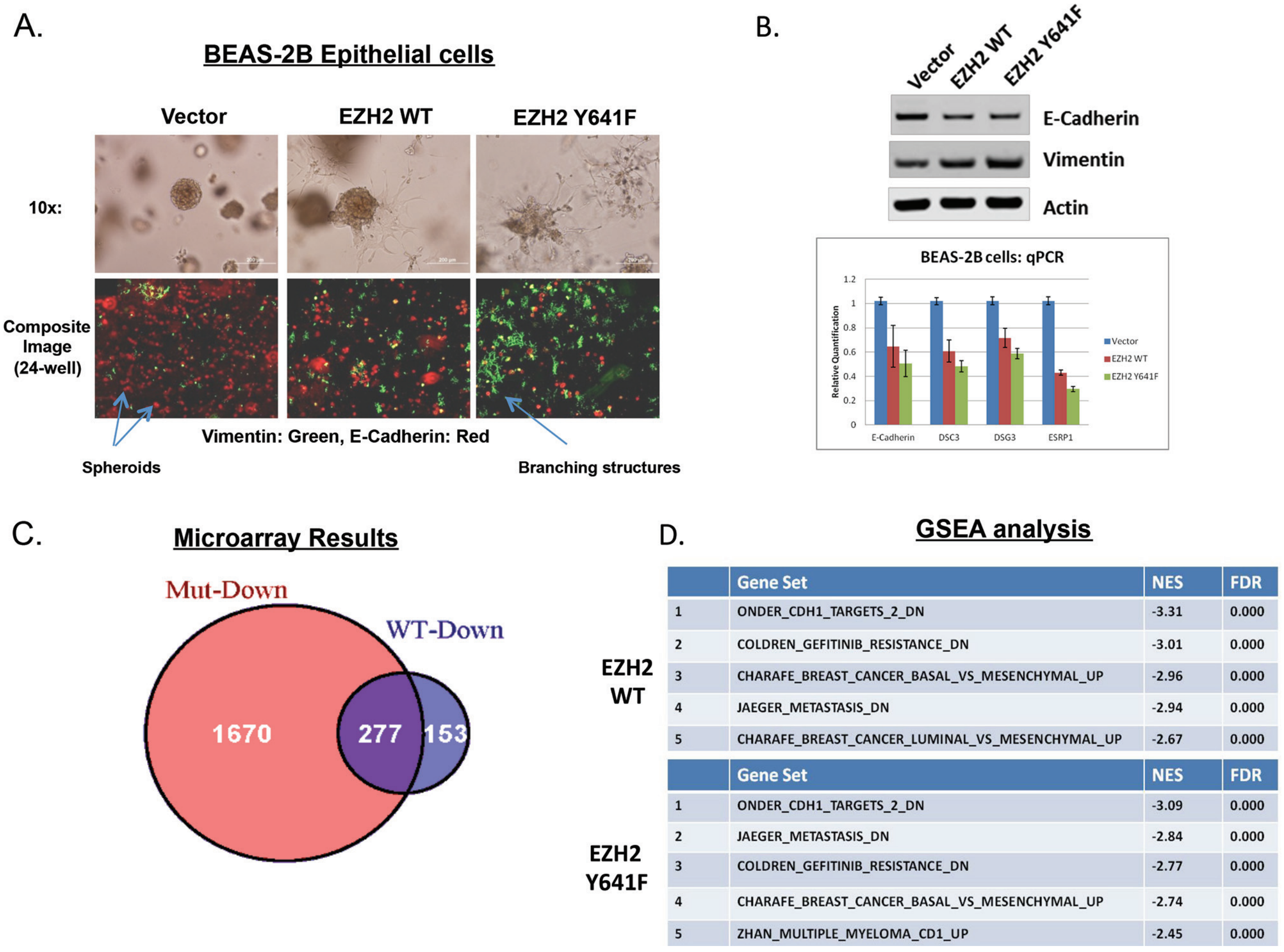

	Gene Set	NES	FDR
1	ONDER_CDH1_TARGETS_2_DN	-3.31	0.000
2	COLDREN_GEFITINIB_RESISTANCE_DN	-3.01	0.000
3	CHARAFE_BREAST_CANCER_BASAL_VS_MESENCHYMAL_UP	-2.96	0.000
4	JAEGER_METASTASIS_DN	-2.94	0.000
5	CHARAFE_BREAST_CANCER_LUMINAL_VS_MESENCHYMAL_UP	-2.67	0.000

	Gene Set	NES	FDR
1	ONDER_CDH1_TARGETS_2_DN	-3.09	0.000
2	JAEGER_METASTASIS_DN	-2.84	0.000
3	COLDREN_GEFITINIB_RESISTANCE_DN	-2.77	0.000
4	CHARAFE_BREAST_CANCER_BASAL_VS_MESENCHYMAL_UP	-2.74	0.000
5	ZHAN_MULTIPLE_MYELOMA_CD1_UP	-2.45	0.000

Supplementary Figure S3: EZH2 GOF causes features of the epithelial to mesenchymal transition (EMT) in BEAS-2B cells. (A) Top panel shows 10x images of BEAS-2B cells in 3D-culture (15 days). Bottom panel shows composite images covering the majority of one-well of a 24-well plate. Staining is shown for E-Cadherin (red) or Vimentin (green). **(B)** Top panel: Western blot analysis was performed for two classic EMT markers (E-Cadherin and Vimentin) following expression of an empty vector, EZH2 WT or EZH2 Y641F in BEAS-2B cells. Actin serves as a loading control. Bottom panel: qRT-PCR analysis of BEAS-2B stable cells is shown for E-Cadherin and additional genes that are markers of the epithelial status (DSC3= Desmocollin 3, DSG3= Desmoglein 3, ESRP= epithelial splicing regulatory protein 1, (mean −/+ STDEV)). **(C)** The Venn diagram (bottom left panel) gives the number of qualifiers down-regulated by either EZH2 GOF or WT as assessed by microarray analysis. D. GSEA analysis was performed from data obtained by microarray analysis. The top down-regulated gene sets are shown for EZH2 WT and Y641F.

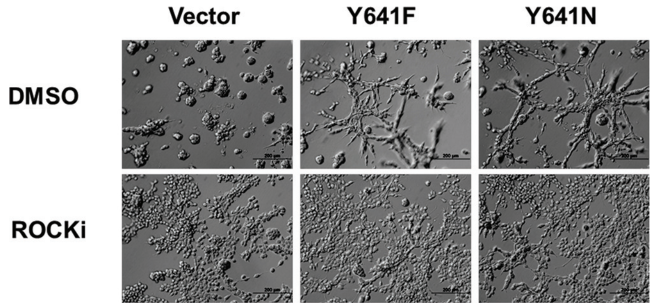

Supplementary Figure S4: Long term ROCK inhibition causes A375 cells to form large cell clusters devoid of branching structures in 3D-culture. A375 Vector, Y641F and Y641N expressing cells were simultaneously treated or untreated with 10 µM Y-27632 (ROCK inhibitor) and plated on top of ECM. Images (10x) were captured 4 days later.

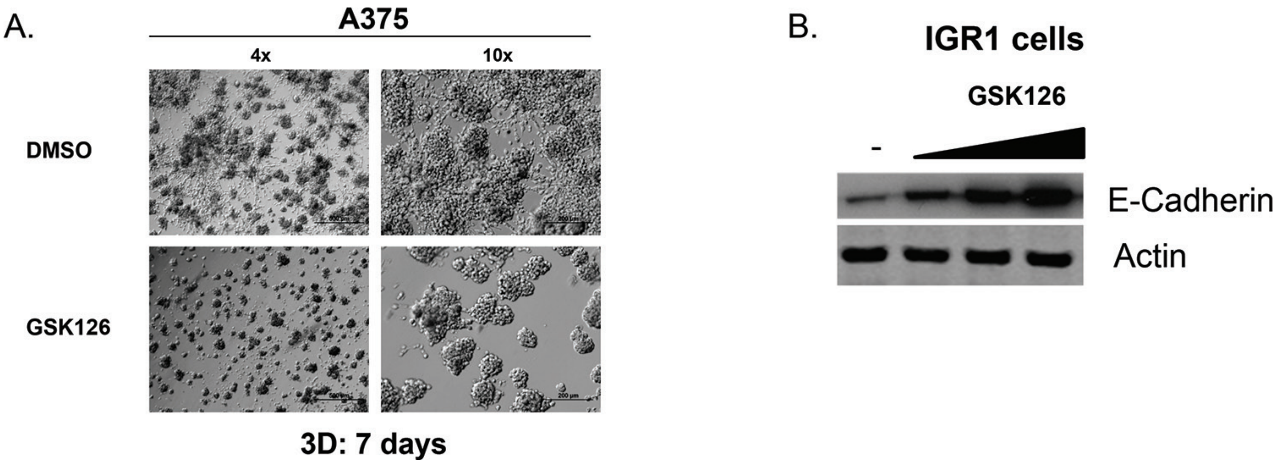

Supplementary Figure S5: Catalytic inhibition of endogenous EZH2 alters 3D morphology and target gene expression. (A) A375 cells following seven days of 1 µM GSK126-treatment were re-plated (and re-treated) on top of ECM and imaged (4x, 10x) seven days later. **(B)** IGR1 cells were grown on top of ECM for 6 days and lysed for western blot analysis.

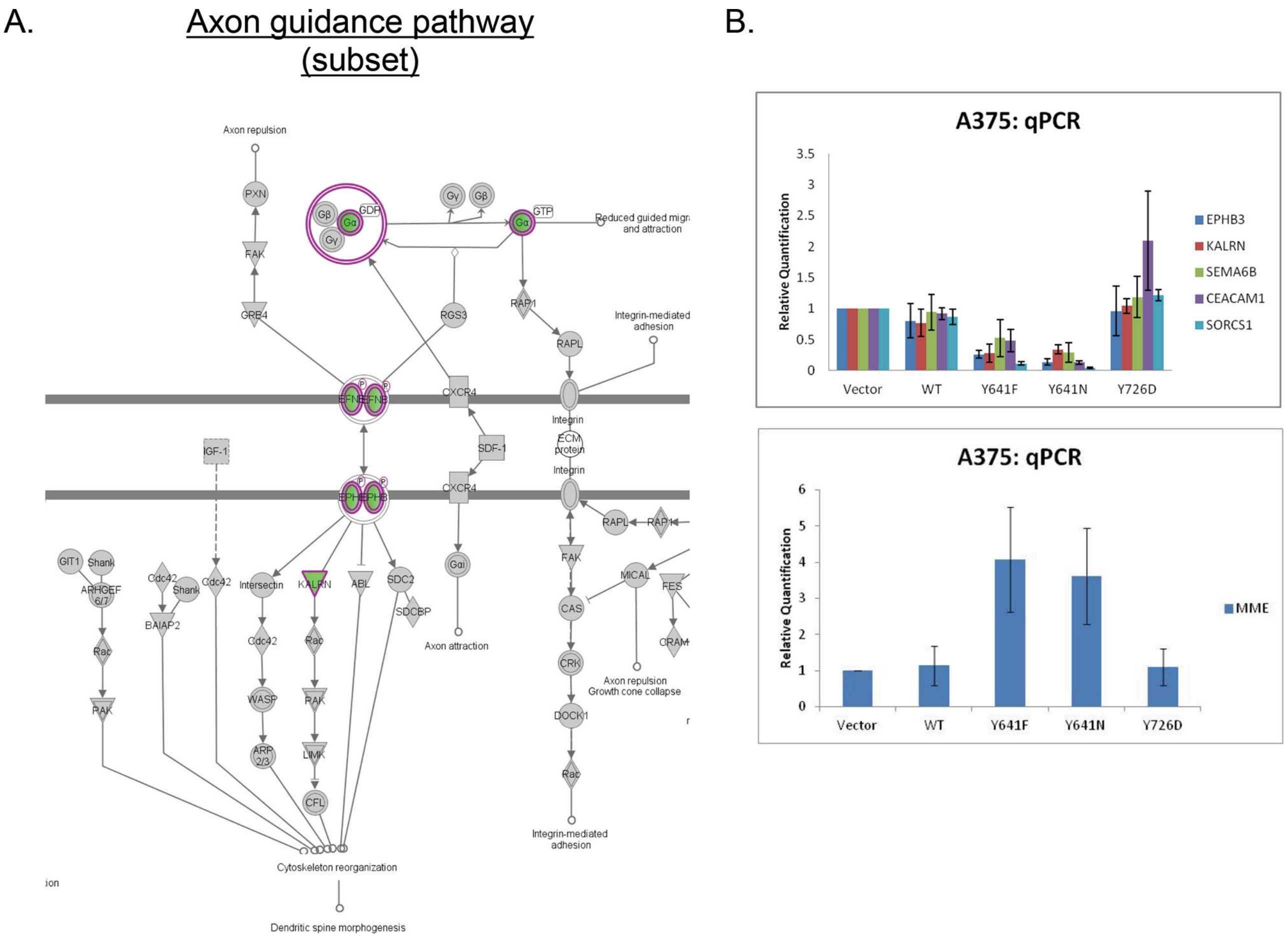

Supplementary Figure S6: EZH2 GOF mutants commonly regulate genes involved in axonal guidance. (A) A small portion of the axonal guidance pathway (IPA analysis) is displayed. Gene expression changes of EZH2 Y641F versus Vector (green signifies down-regulation) are overlaid. **(B)** qPCR was used to validate gene expression changes observed by RNA-seq (mean −/+ STDEV).

Supplementary Table S1: Microarray analysis of BEAS-2B cells.

Supplementary Table S2: RNA-seq analysis of A375 cells and IGR1 cells.

SUPPLEMENTARY MOVIE FILES

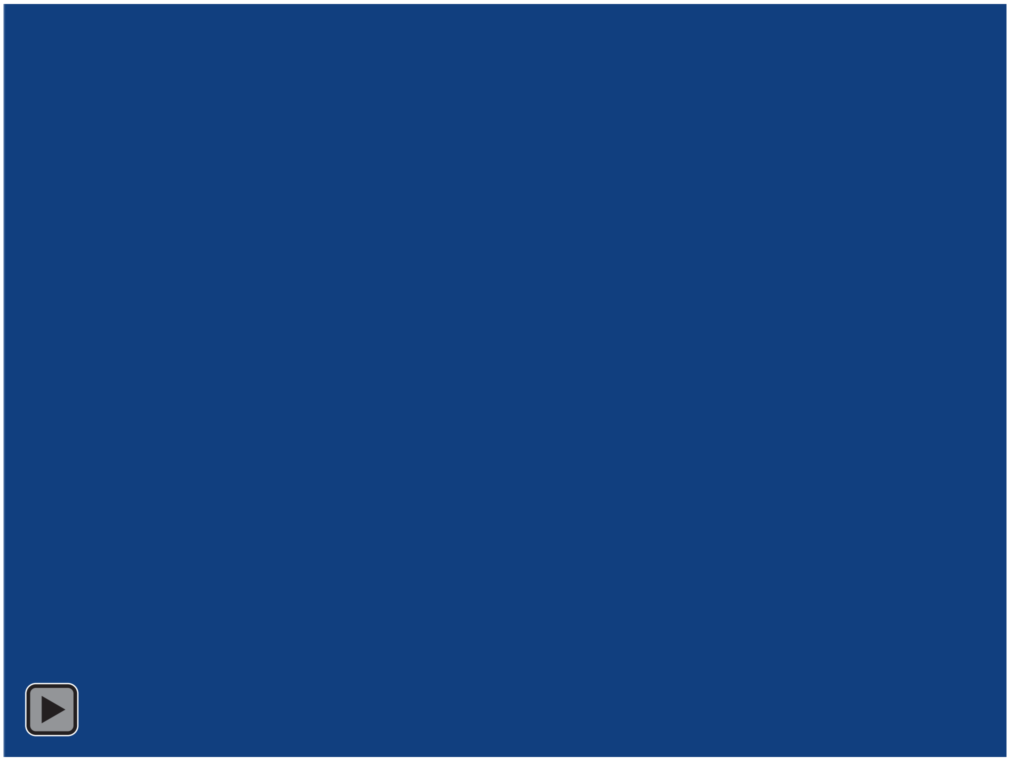

Supplementary Video S1: A375 Vector (Control).

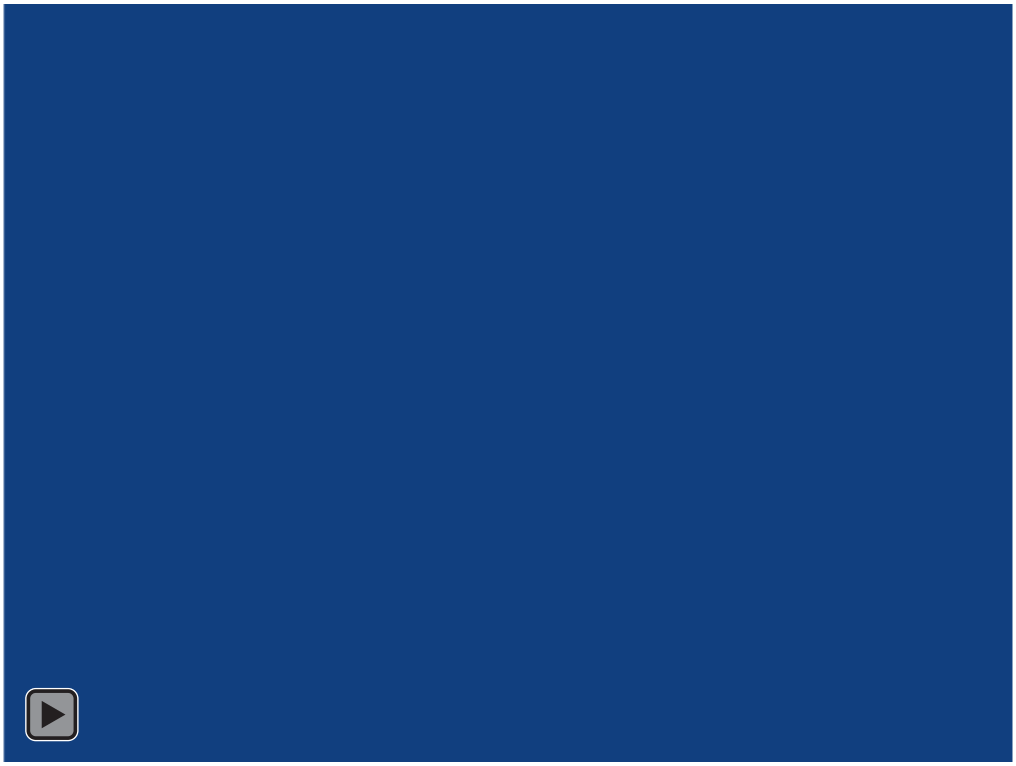

Supplementary Video S2: A375 EZH2 WT.

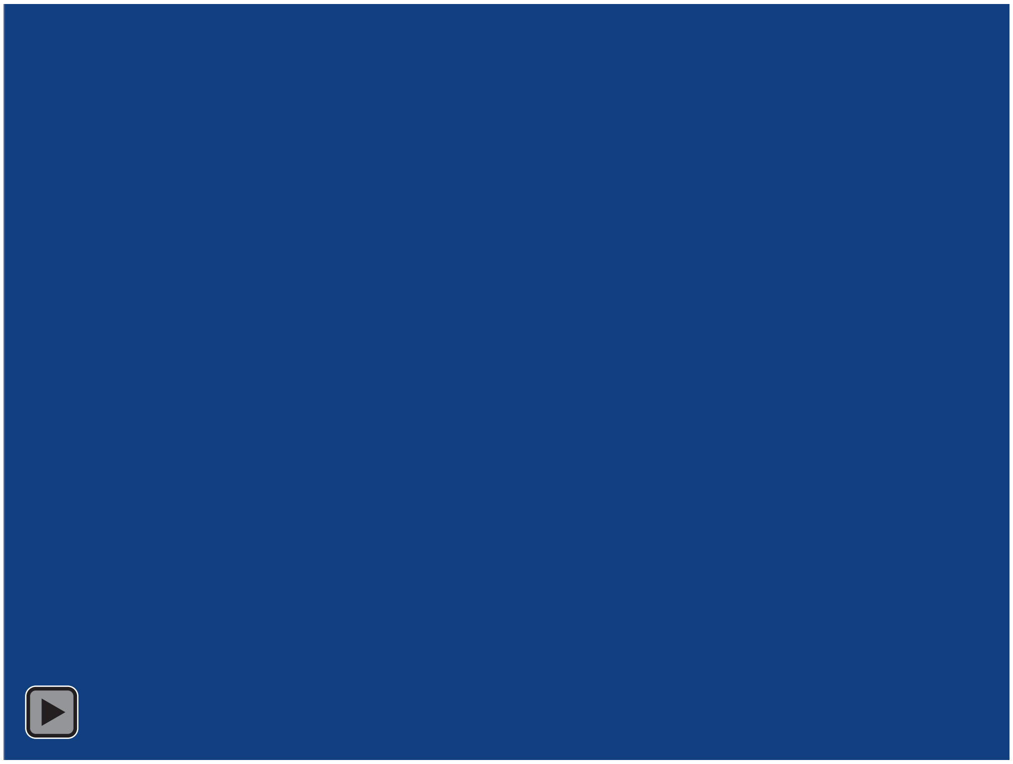

Supplementary Video S3: A375 EZH2 Y641F (GOF).

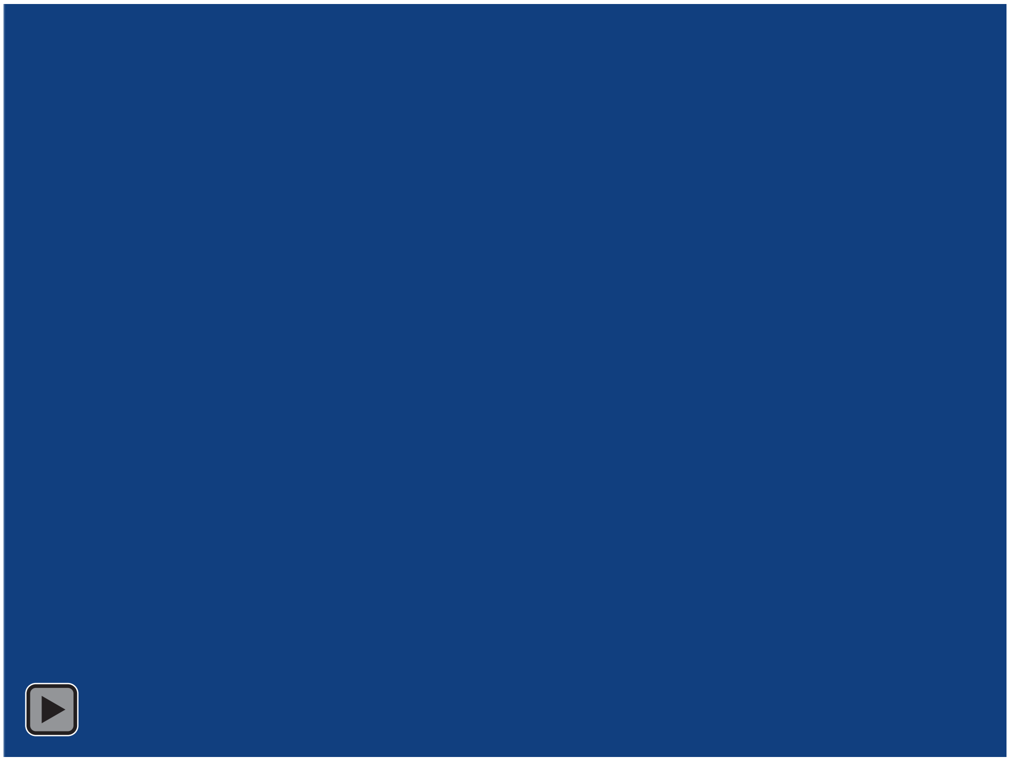

Supplementary Video S4: A375 EZH2 Y641N (GOF).

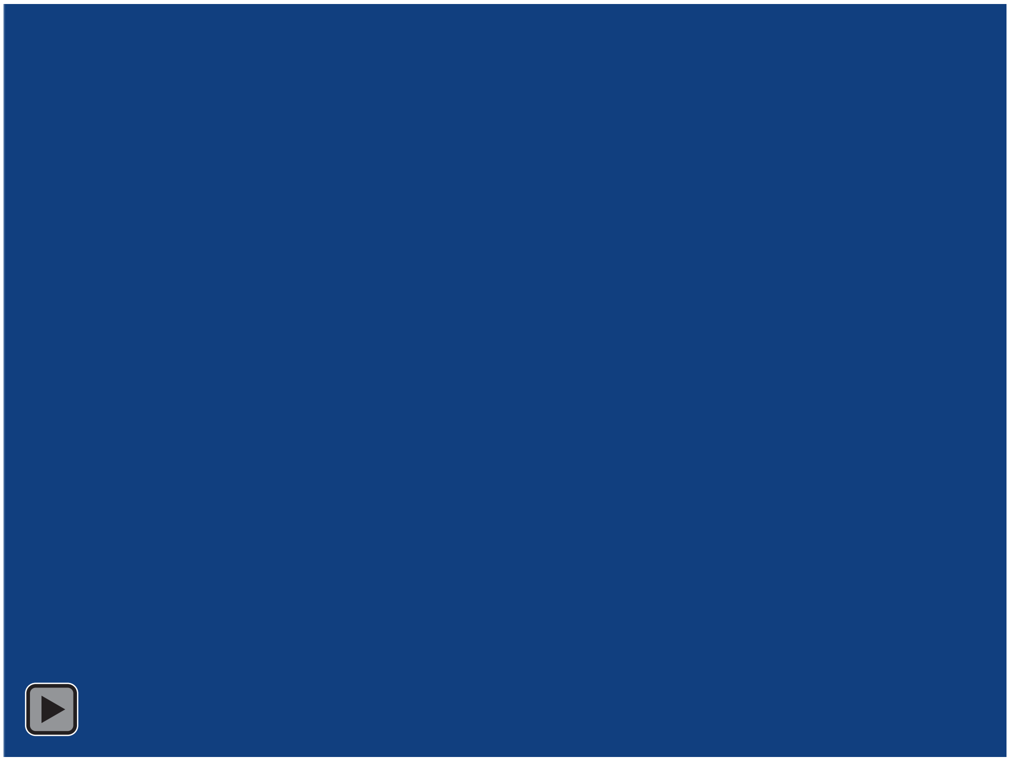

Supplementary Video S5: A375 EZH2 Y726D (LOF).

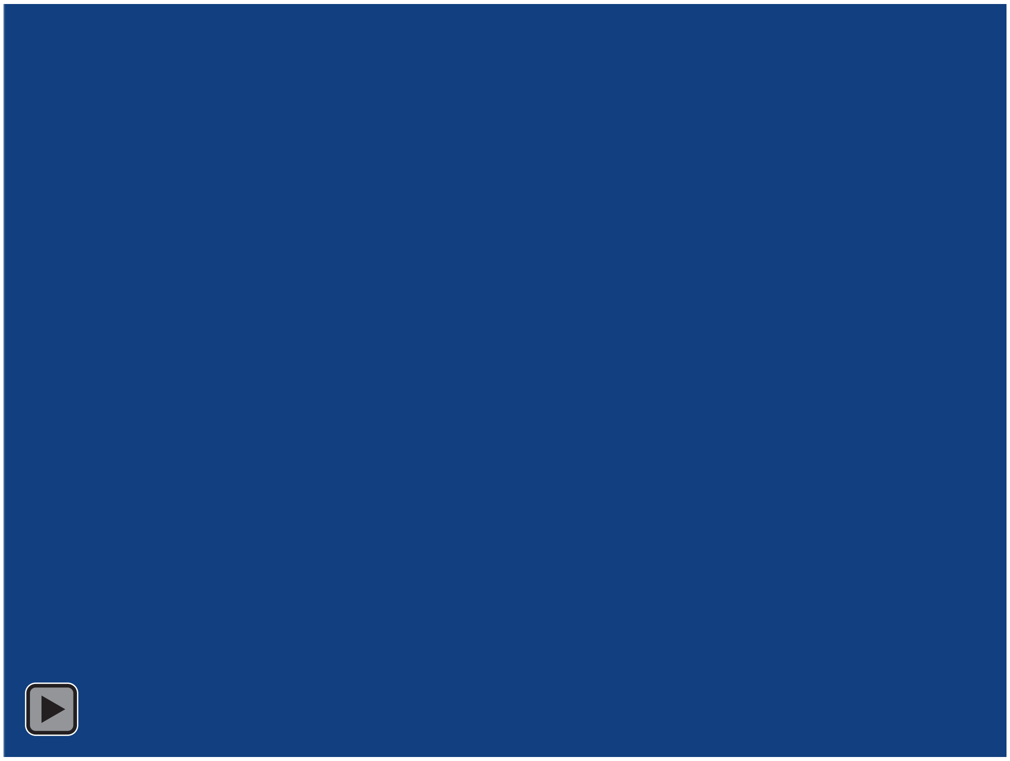

Supplementary Video S6: A375 Vector + ROCK inhibitor.
